# Considerations for the Use of Glucagon-like Peptide-1 Medications for Obesity in a Plastic Surgery Setting

**DOI:** 10.1097/GOX.0000000000007085

**Published:** 2025-09-10

**Authors:** Leah M. Schumacher, David B. Sarwer

**Affiliations:** From the Department of Social and Behavioral Sciences, Center for Obesity Research and Education, College of Public Health, Temple University, Philadelphia, PA.

## Abstract

In recent years, there has been great interest among the medical community and general public in a new generation of medications for treating obesity. Likely due to this interest, there have been anecdotal reports of plastic surgeons beginning to prescribe these medications in their practices. This Special Topic article provides a brief overview of obesity and its evidence-based treatments, including these newer medications. Particular emphasis is placed on the growing consensus that obesity is a chronic disease that contributes to numerous other comorbidities and requires sustained treatment. Plastic surgeons who are interested in using newer obesity medications in their practices may wish to consider the need for additional training to ensure that they are optimizing patient welfare, guarding against harm, and providing care consistent with clinical guidelines and recommended best practices of obesity medicine.

**Takeaways**

**Question:** Are plastic surgeons beginning to prescribe newer generation obesity medications (eg, glucagon-like peptide-1 agonists) in their practices, as suggested by anecdotal reports?

**Findings:** This article discusses important considerations for plastic surgeons who are considering offering these medications in their practices or already doing so.

**Meaning:** Plastic surgeons who are interested in using newer obesity medications may wish to consider the need for additional training to ensure that they are optimizing patient welfare, guarding against harm, and providing care consistent with clinical guidelines and recommended best practices of obesity medicine.

## INTRODUCTION

The prevalence of obesity has grown steadily during the past several decades, arguably making it one of the world’s most pressing public health issues.^1,2^ More than 40% of the adult population of the United States now has obesity, operationalized as a body mass index (BMI) of 30 kg/m^2^ or more.^3^ Another third of the population has a BMI in the overweight range (BMI > 25 kg/m^2^),^4^ leaving these individuals at risk for the development of obesity as well as weight-related health problems, such as type 2 diabetes, cardiovascular disease, and several forms of cancer.^5–8^ Excess body weight represents a significant risk to overall health and life expectancy, despite the long-standing and still enduring belief that excess body weight is primarily an aesthetic concern.

There are 3 main categories of evidence-based obesity treatments.^5^ These include lifestyle modification approaches (reducing caloric intake, increasing physical activity, and behavioral modification), obesity medications (often colloquially referred to as weight loss medications), and metabolic and bariatric surgery.^5,9^ Each of these treatments can result in clinically significant reductions in weight, often defined as a loss of 5%–10% or more of initial body weight, as well as improvements in weight-related comorbidities.^5^

Several new obesity medications have received Food and Drug Administration (FDA) approval in recent years.^10^ These medications are often referred to as GLP-1s (glucagon-like peptide-1 receptor agonists, eg, semaglutide) or GLP-1 dual agonists (agonists of GLP-1 and glucose‐dependent insulinotropic polypeptide, eg, tirzepatide). Table 1 provides an overview of the medications that currently have FDA approval^11–16^; more detailed discussions of these medications, their mechanisms of action, and the most common side effects can be found elsewhere.^17–20^

**Table 1.** Overview of FDA-approved Medications for the Long-term Treatment of Obesity

Generic Name	Brand Name	Indications	Route of Administration	Dosage Guidelines for Adults	Expected Weight Loss
Orlistat	Xenical, Alli	• Overweight or obesity in individuals ≥12 y^*^^,^^†^^,^^‡^	Pill taken by mouth	120 mg 3x/d	~10% at 12 mo^11^
Phentermine–topiramate	Qsymia	• Overweight or obesity in individuals ≥12 y^*^^,^^†^	Pill taken by mouth	1x/d Week 1–2: 3.75 mg/23 mg; week 3–12+: 7.5 mg/46 mg; week 13–14^§^: 11.25 mg/69 mg; week 15+^§^: 15 mg/92 mg	~11% at 12 mo^12^
Bupropion–naltrexone	Contrave	• Overweight or obesity in individuals ≥18 y^*^	Pill taken by mouth	1–2x/d Week 1: 8 mg/90 mg qd; week 2: 8 mg/90 mg bid; week 3: 16 mg/180 mg qd, 8 mg/90 mg qd; week 4: 16 mg/180 mg bid	~6% at 12 mo^13^
Liraglutide	Saxenda (brand name for type 2 diabetes: Victoza)	• Overweight or obesity in individuals ≥12 y^*^^,^^¶^	Subcutaneous injection	1x/d Week 1: 0.6 mg qd; week 2: 1.2 mg qd; week 3: 1.8 mg qd; week 4: 2.4 mg qd; week 5+: 3.2 mg qd	~8% at 12 mo^14^
Semaglutide	Wegovy (brand name for type 2 diabetes: Ozempic, Rybelsus)	• Overweight or obesity in individuals ≥12 y^*^^,^^†^ • Reduce risk of serious cardiovascular events in adults with cardiovascular disease and overweight/obesity	Subcutaneous injection	Weekly Week 1–4: 0.25 mg/wk; week 5–8: 0.5 mg/wk; week 9–12: 1.0 mg/wk; week 13–16+: 1.7 mg/wk; week 17 + ^§^: 2.4 mg/wk	~15% at 16 mo^15^
Tirzepatide	Zepbound (brand name for type 2 diabetes: Mounjaro)	• Overweight or obesity in individuals ≥18 y^*^ • Moderate-to-severe OSA in adults with obesity	Subcutaneous injection	Weekly Week 1–4: 2.5 mg/wk; week 5–8 + ^§^: 5 mg/wk; week 9–12^§^: 7.5 mg/wk; week 13–16+^§^: 10 mg/wk; week 17–20^§^: 12.5 mg/wk; week 21+^§^: 15 mg/wk Recommended maintenance doses: weight management—5, 10, and 15 mg; OSA—10 and 15 mg	~21% at 17 mo^16^

Information shown is for obesity formulations only. Expected weight loss values are for the maximum approved dosage. Information about setmelanotide, an approved medication for obesity due to specific rare, genetic conditions, is not included.

* Approved for adults with a BMI ≥ 30 kg/m^2^ (obesity) or a BMI ≥ 27 kg/m^2^ (overweight) with at least 1 weight-related condition.

† Approved for adolescents 12–17 years with a BMI ≥ 95th percentile.

‡ Alli only approved for adults ≥18 years and approved for use in BMI ≥ 25 kg/m^2.^

§ As needed, based on initial weight loss and treatment goals.

¶ Approved for adolescents 12–17 years with a BMI cutoff for age and sex corresponding to a BMI ≥ 30 kg/m^2^ for adults and who weigh >60 kg.

OSA, obstructive sleep apnea.

In general, the GLP-1s produce greater weight losses than those typically achieved with previous medications with FDA approval or lifestyle modification.^20^ For example, individuals prescribed the drug semaglutide plus a lifestyle modification intervention for 68 weeks lost an average of 15% of their body weight.^15^ In a trial of tirzepatide, the average weight loss at 72 weeks was 15%–21%, depending on the dose, and more than two-thirds of patients lost at least 10% of their body weight.^16^

A rapidly growing research base also indicates that these weight losses are associated with profound improvements in common and serious comorbidities, including type 2 diabetes, kidney disease, cardiovascular disease, and nonalcoholic fatty liver disease.^21–23^ Consequently, these medications have garnered significant media attention and have quickly made their way into the awareness of many Americans. For example, a nationally representative poll conducted in May 2024 found that 82% of Americans had heard at least a little about these medications, and 1 in 8 Americans had tried a GLP-1.^24,25^

For many individuals, the primary motivation to lose weight is aesthetic.^26^ This focus on losing weight to improve appearance is understandable given strong cultural beauty ideals emphasizing thinness. However, the academic and obesity medicine communities’ understanding of obesity has evolved quite markedly in recent years. For example, the American Medical Association’s official recognition of obesity as a disease in 2013 reflects advancements in understanding of both the complex etiology of obesity and the need for comprehensive management of obesity to yield sustained, positive outcomes.^27^ A recent report in *The Lancet*, focused on establishing objective criteria for defining and diagnosing clinical obesity as a chronic disease while increasing emphasis on the major comorbidities of excess body weight and de-emphasizing BMI, echoes the importance of seeing obesity as a disease that requires thorough assessment (often by a multidisciplinary team) and ongoing treatment.^28^

As awareness of and consumer demand for GLP-1 medications has increased, there have been anecdotal reports of plastic surgeons beginning to prescribe GLP-1s.^29,30^ Although several papers have been written for plastic surgeon audiences that provide primers on these medications, discuss perioperative safety considerations, or discuss considerations for certain procedures (eg, body contouring, facial rejuvenation),^30–33^ empirical data on rates of usage of GLP-1s in plastic surgery settings are both lacking and needed. Further, little attention has been given to changes to clinical practices that may need to occur so that plastic surgeons and team members can appropriately assess and treat patients with obesity and its related comorbidities. These considerations, along with recommendations to address them, are discussed in the following sections.

Patients who seek GLP-1 prescriptions from plastic surgeons may not appreciate the complexities of obesity and the impact that excess weight can have on more than 200 additional health conditions.^34^ As a result, they may not have been screened for the comorbidities often seen with obesity, such as obstructive sleep apnea and nonalcoholic fatty liver disease.^35,36^ Plastic surgeons may also be unfamiliar with the relationship between obesity and these, as well as other, comorbidities and thus may not assess them in the same way as internal medicine physicians, who are the most frequent prescribers of the GLP-1s.^37^

Patients and surgeons who view obesity largely as an aesthetic concern, and not a medical one, may have expectations for treatment outcomes not grounded in the current evidence base. For example, patients may expect weight loss in certain “problem” areas. There is no evidence to suggest that the medications accomplish this. Further, there is evidence to suggest that some patients on these medications lose a sizable percentage of lean muscle mass, which can impact body shape.^38^ There have been anecdotal reports, largely on social media, of patients and providers “microdosing” these medications for weight maintenance or to promote small amounts of weight loss (eg, 10–20 lbs) among patients who do not have obesity. There is currently no evidence-based protocol for this practice. Patients also may not appreciate that these medications must be used long-term for sustained benefit.^39,40^ Maximum weight losses are reached after 12–18 months of continuous use for most individuals, with discontinuation being associated with weight regain for many.^39,40^

If plastic surgeons are going to provide prescriptions for these medications, it is important that they are prepared to help patients shift from a primarily aesthetic view of obesity to a more holistic, disease-oriented view. This can position patients to better understand the impact of their excess body weight on organ systems throughout the body, seek out appropriate assessment and treatment as needed, and adopt a more realistic and appropriate long-term view of obesity management. Patients and providers should understand that the current generation of obesity medications was tested in and is clinically indicated for a select population—individuals with excess adiposity who are at elevated risk for or already experiencing negative obesity-related health effects. The medications have not been tested, nor do they have FDA approval, for persons considered overweight and metabolically healthy.

The 2025 report from the Commission on the Definition and Diagnosis of Clinical Obesity published in *Lancet Diabetes & Endocrinology* provides detailed guidance for how to determine who has clinical obesity and warrants treatment with pharmacological agents.^28^ Various other clinical guidelines also provide insights into how to assess patients’ weight-related health and determine when these medications are appropriate.^41–44^ The medications are contraindicated for certain individuals, such as patients with certain gastrointestinal conditions and those who are pregnant or breastfeeding.^10^ As a result, a thorough history and medical assessment is essential to ensure appropriate prescribing. Additionally, these medications affect the rate of gastric emptying, which may thereby affect the bioavailability of a number of medications.^45^ Dosing of other medications the patient is taking may be weight-dependent; as patients lose weight, doses of medications for hypertension and type 2 diabetes may need to be adjusted. The physician prescribing the anti-obesity medication, the plastic surgeon in this case, typically assumes the responsibility of recommending or making changes to those medications, something that may not be a matter of routine in many plastic surgery practices.

If obesity medications are prescribed, there is a need for ongoing monitoring and care coordination to ensure that medications are effective and well tolerated. Appropriate counseling of patients at the time of prescription and ongoing monitoring can also help to prevent and monitor side effects.^10^ Coverage from insurance plans may require that patients are seen in clinic on a monthly basis to document sufficient weight loss and ensure patient safety with regard to improvements in comorbidities that may require changes in dosages of medications.^46^ As noted earlier, this frequency of patient–provider contact may not be the norm in many plastic surgery practices. Plastic surgeons also need to be prepared to help patients with long-term weight maintenance, over years and not months, lest their efforts at treating obesity are just added to the patient’s list of previously tried approaches yielding less-than-desired results.

Plastic surgeons should also be mindful of the psychosocial burden of obesity that many patients experience.^47^ Weight stigma, which refers to negative attitudes, stereotypes, or discriminatory acts toward individuals based on their weight, is commonly reported by patients with obesity.^48^ Stigmatizing views are unfortunately common among healthcare providers and are associated with greater psychosocial distress and physical disease burden.^48,49^ Providers can ensure that they are not furthering weight stigma or unintentionally offending patients by educating themselves about weight stigma, reflecting on their own attitudes and behaviors toward persons with obesity, and adopting best practices for stigma-free care.^50,51^

Most physicians across the medical specialties receive little training on obesity during medical education.^52,53^ Even providers who are regularly engaged in obesity management, such as primary care physicians and endocrinologists, report knowledge gaps about GLP-1s and dual agonists.^54^ Fortunately, there are now many resources available to providers who want to improve their obesity medicine knowledge and skills. These range from 1-time seminars to board certification in obesity medicine, with board certification being the most robust training.^55,56^ Plastic surgeons interested in delivering the highest quality care for obesity are encouraged to participate in these educational activities.^56^ Even plastic surgeons who are not interested in offering these treatments may benefit from continuing education on obesity, as this will allow them to better understand their patients’ weight-related concerns and provide more appropriate referrals to evidence-based treatment.

Obesity affects millions of adults, and rates are expected to rise.^1,57^ The scope of the disease warrants an “all hands on deck” approach to treating patients. Plastic surgeons can contribute to these efforts, especially given the effectiveness and scalability of GLP-1s and the likely emergence of other highly effective medications in the future. If plastic surgeons are interested in using these medications in their practices, we encourage them to adopt a chronic disease view of obesity and to approach treatment thoughtfully and comprehensively. Doing so is in the true spirit of high-quality, holistic, patient-centered care.

## DISCLOSURES

Dr. Schumacher reports funding from the National Institutes of Health (National Heart, Lung, and Blood Institute, L30 HL154167). Dr. Sarwer has grant funding from the National Institutes of Health (National Institute for Diabetes, Digestive, and Kidney Disease, R01 DK108628 and R01 DK133264). He is a Consulting Editor for Plastic and Reconstructive Surgery.
